# Abrogation of Nrf2 impairs antioxidant signaling and promotes atrial hypertrophy in response to high-intensity exercise stress

**DOI:** 10.1186/s12967-016-0839-3

**Published:** 2016-04-05

**Authors:** Radhakrishnan Rajesh Kumar, Madhusudhanan Narasimhan, Gobinath Shanmugam, Jennifer Hong, Asokan Devarajan, Sethu Palaniappan, Jianhua Zhang, Ganesh V. Halade, Victor M. Darley-Usmar, John R. Hoidal, Namakkal S. Rajasekaran

**Affiliations:** Cardiac Aging & Redox Signaling Laboratory, Division of Molecular & Cellular Pathology, Department of Pathology, The University of Alabama at Birmingham, Birmingham, AL 35294-2180 USA; Department of Pharmacology and Neuroscience, Texas Tech University Health Sciences Center, 3601 4th Street, Lubbock, TX 79430 USA; Division of Cardiovascular Medicine, Department of Medicine, The University of Utah School of Medicine, Salt Lake City, UT 84132 USA; Department of Obstetrics and Gynecology, David Geffen School of Medicine at UCLA, Los Angeles, CA 90095 USA; Department of Bio-Engineering, Comprehensive Cardiovascular Center, The University of Alabama at Birmingham, Birmingham, AL USA; Center for Free Radical Biology, The University of Alabama at Birmingham, Birmingham, AL 35294-2180 USA; Department of Medicine, Comprehensive Cardiovascular Center, The University of Alabama at Birmingham, Birmingham, AL USA; Division of Pulmonary Medicine, Department of Medicine, The University of Utah School of Medicine, Salt Lake City, UT 84132 USA; Department of Exercise Physiology, College of Health, The University of Utah School of Medicine, Salt Lake City, UT 84132 USA

**Keywords:** Nrf2 knockout, Autophagy, Atrial hypertrophy, Antioxidants, Oxidative stress

## Abstract

**Background:**

Anomalies in myocardial structure involving myocyte growth, hypertrophy, differentiation, apoptosis, necrosis etc. affects its function and render cardiac tissue more vulnerable to the development of heart failure. Although oxidative stress has a well-established role in cardiac remodeling and dysfunction, the mechanisms linking redox state to atrial cardiomyocyte hypertrophic changes are poorly understood. Here, we investigated the role of nuclear erythroid-2 like factor-2 (Nrf2), a central transcriptional mediator, in redox signaling under high intensity exercise stress (HIES) in atria.

**Methods:**

Age and sex-matched wild-type (WT) and Nrf2^−/−^ mice at >20 months of age were subjected to HIES for 6 weeks. Gene markers of hypertrophy and antioxidant enzymes were determined in the atria of WT and Nrf2^–/–^ mice by real-time qPCR analyses. Detection and quantification of antioxidants, 4-hydroxy-nonenal (4-HNE), poly-ubiquitination and autophagy proteins in WT and Nrf2^−/−^ mice were performed by immunofluorescence analysis. The level of oxidative stress was measured by microscopical examination of di-hydro-ethidium (DHE) fluorescence.

**Results:**

Under the sedentary state, Nrf2 abrogation resulted in a moderate down regulation of some of the atrial antioxidant gene expression (*Gsr*, *Gclc*, *Gstα* and *Gstµ*) despite having a normal redox state. In response to HIES, enlarged atrial myocytes along with significantly increased gene expression of cardiomyocyte hypertrophy markers (*Anf*, *Bnf* and β-*Mhc*) were observed in Nrf2^−/−^ when compared to WT mice. Further, the transcript levels of *Gclc*, *Gsr* and *Gstµ* and protein levels of NQO1, catalase, GPX1 were profoundly downregulated along with GSH depletion and increased oxidative stress in Nrf2^−/−^ mice when compared to its WT counterparts after HIES. Impaired antioxidant state and profound oxidative stress were associated with enhanced atrial expression of LC3 and ATG7 along with increased ubiquitination of ATG7 in Nrf2^−/−^ mice subjected to HIES.

**Conclusions:**

Loss of Nrf2 describes an altered biochemical phenotype associated with dysregulation in genes related to redox state, ubiquitination and autophagy in HIES that result in atrial hypertrophy. Therefore, our findings direct that preserving Nrf2-related antioxidant function would be one of the effective strategies to safeguard atrial health.

## Background

Heart failure is a leading health problem with over 500,000 new cases and ~300,000 deaths annually in the United States [1]. The pathophysiology of heart and cardiovascular diseases such as heart failure, coronary heart disease, cardiomyopathy, fibrosis, ventricular hypertrophy, atrial fibrillation etc. is closely connected to redox imbalance [2, 3]. Oxidative changes not only prompt molecular conformation of proteins, lipids, and nucleic acids but also elaborate the signals involved in the propagation of structural remodeling of cells and tissues. In relevance, the burst of oxidative species generated from cardiomyocytes and endothelial cells magnifies the inflammatory signals and together they commence a cascade of downstream events [4]. These signaling events, in turn, affect the cardiac structures and electrical modeling thus initiating, steering and/or promoting atrial remodeling [5]. Of note, this remodeling could either be adaptive or maladaptive regulation of cardiac myocytes and other cells in the atria that is dependent on the length of exposure to oxidative stress. The significance and pathogenic consequences of chronic oxidative stress (COS) and ventricular dysfunction in failing hearts have received much clinical attention [6, 7]. Interestingly, some studies indicate that atrial changes could be an important causal mechanism for ventricular dysfunction since ~20 % of left ventricular stroke volume at rest is attributed to atrial contraction [2, 7–9]. It has been described that atrioventricular interaction is in a vicious cycle, i.e., change in atria can affect ventricle and vice versa [10]. Thus, a maladaptive atrial structural change could serve as a central cause for a spectrum of pathophysiological processes associated with cardiac failure [11]. In addition, the limited efficacy and serious off-target adverse effects challenge the current therapeutic management of atrial remodeling. Despite an existence of a clear association between oxidative stress and atrial pathophysiological changes [12], the mechanisms underlying the atrial remodeling is still poorly understood.

Transcriptional regulation of antioxidant genes by specific endogenous factors has been gaining increasing attention [13, 14]. Of interest, Nrf2 has been recognized as a master transcriptional regulator of antioxidant genes [15, 16]. Activated Nrf2 binds to the antioxidant/electrophile response element (ARE/EpRE) in the promoter of target antioxidant genes and regulates their transcription [17]. Numerous studies have demonstrated that loss and/or dysregulation of Nrf2 are often linked with various diseases [18–21]. Davies and co-workers have demonstrated that habitual exercise training decreases oxidative stress [22–24] and recent studies from our laboratory has shown that acute exercise activates Nrf2/ARE signaling promoting the antioxidant defense genes in the myocardium [25]. However, the role of Nrf2 signaling in response to high endurance activity in the atria has not been investigated. In eukaryotic cells, autophagy mechanism is typically used to deliver cytoplasmic proteins and organelles to lysosomes [26, 27]. It is augmented during cell growth, development, and death and has been associated with several pathological conditions such as cancer, neurodegenerative disorders, myopathies, heart and liver diseases, and gastrointestinal disorders [28]. However, the autophagic activity is shown to decline with increasing age that in turn leads to dampened response to stress and thus, inefficient clearance of damaged proteins and organelles in cells and tissues [29]. Herein, the autophagic response was estimated in terms of measuring the levels of LC3, an important regulator that initiates autophagosome biogenesis and Atg7, a key autophagy promoting molecule [30].

In this study, our goal was to examine the effect of high-intensity exercise stress (HIES) on redox-sensitive, Nrf2 signaling in atria of age and sex matched >20 months old wild-type (WT) and mice genetically engineered to lack Nrf2 (Nrf2^−/−^). The study tests the hypothesis that genetic disruption or abrogation of Nrf2 in association with high-intensity exercise stress (HIES) could induce atrial remodeling on aging. Given a high predictive value of oxidative stress markers in myocardial injury, fibrosis and cardiac muscle damage as well as a wide knowledge gap with respect to mechanistic understanding of pathological remodeling, our current study will present the biochemical basis of Nrf2 signaling mechanism in the process of atrial cardiomyocyte remodeling that ensue with age-associated oxidative challenge [31]. Further, it will enhance our ability to target the Nrf2 signaling as a potential therapeutic direction and help develop therapeutic agents to control the pathogenesis associated with aging atria.

## Methods

### Animals

C57/BL6/SJ wild-type (WT) and Nrf2 knock out (Nrf2^−/−^) (C57/BL6/SJ background) mice (>20 months of age) were used to study the effect of redox imbalances in atria. Mice were obtained from Dr. Li Wang (University of Utah, USA). The animals were maintained in the Small Animal Housing and Research Facility at University of Utah. Mice were housed not more than n = 5/cage with free access to food (standard rodent diet) and water ad libitum and maintained under conditions controlled for temperature and humidity, artificial lighting for 12-h from 6 AM to 6 PM light/dark cycle. The animal exercise and usage protocols were approved by Institutional Animal Care and Use Committee (IACUC) of University of Utah and then by the University of Alabama at Birmingham.

### High-intensity exercise program

Wild-type and Nrf2^−/−^ mice (n  =  3–4/group/experiment) were randomly assigned for high-intensity exercise (HIES) on a treadmill as described previously [32]. The HIES protocol was developed based on our previous exercise protocols ranging from acute-moderate, acute-endurance, short-term moderate and long-term endurance regimen [18, 25, 32, 33]. Briefly, HIES mice were run on the treadmill for 6 weeks at 20–25 m/min; 12 % grade for 60 min per day. Before subjecting the mice for HIES, they were trained for 1 week on the treadmill for 10 min; 15–22 m/min; 0–12 % grade to check their endurance ability. Equal numbers of mice were assigned to the sedentary group and were not subjected to HIES. After 6 weeks of HIES, mice were sacrificed by CO_2_ inhalation and tissues were harvested after 3 h following the last bout of exercise. Left and right atria were carefully dissected out by removing ventricles, aorta and fat bodies using a dissection microscope. Then the atria were stored either in RNA later (Sigma) followed by frozen at −80 °C or embedded in O.C.T media and flash frozen in liquid nitrogen and stored for subsequent analyses.

### RNA isolation, reverse transcription and qRT-PCR

Atrial RNA from WT and Nrf2^−/−^ mice (HIES and sedentary) was extracted using Qiagen kit (74106) following the manufacturer’s instructions. 1.25 μg of RNA was used to synthesize cDNA (Qiagen Reverse Transcription Kit #205311) and 25–50 ng of cDNA template in a final reaction mixture of 10 µL of SYBR green master mix (Qiagen #204054) and respective primer sets (1 pmol) (Table 1) was PCR amplified in a Light Cycler real-time thermocycler (Roche Light Cycler-480). Quantification of mRNA was done using Ct values, and the target mRNA levels were calculated by normalizing to the Ct values of housekeeping genes Arbp1 or Gapdh by 2^−ΔΔCt^ method [34].

**Table 1:** List of the primers used for the qRT-PCR analysis

Genes name	Sequences (5′…..3′)
*Anf F*	GCTTCCAGGCCATATTGGAG
*Anf R*	GGGGGCATGACCTCATCTT
*Arbp1 F*	TGAGATTCGGGATATGCTGTTGG
*Arbp1 R*	CGGGTCCTAGACCAGTGTTCT
α-*Mhc F*	GAGTGGGAGTTTATCGACTTCG
α-*Mhc R*	CCTTGACATTGCGAGGCTTC
*Bnf F*	GAGGTCACTCCTATCCTCTGG
*Bnf R*	GCCATTTCCTCCGACTTTTCTC
β-*Mhc F*	ACTGTCAACACTAAGAGGGTCA
β-*Mhc R*	TTGGATGATTTGATCTTCCAGGG
*Cat F*	GGAGGCGGGAACCCAATAG
*Cat R*	GTGTGCCATCTCGTCAGTGAA
*Gapdh F*	TGACCTCAACTACATGGTCTACA
*Gapdh R*	CTTCCCATTCTCGGCCTTG
*Gclc F*	GGACAAACCCCAACCATCC
*Gclc R*	GTTGAACTCAGACATCGTTCCT
*Gclm F*	CTTCGCCTCCGATTGAAGATG
*Gclm R*	AAAGGCAGTCAAATCTGGTGG
*Gpx1 F*	CCACCGTGTATGCCTTCTCC
*Gpx1 R*	AGAGAGACGCGACATTCTCAAT
*Gsr F*	CACGGCTATGCAACATTCGC
*Gsr R*	GTGTGGAGCGGTAAACTTTTTC
*Gstm4 F*	CTGAAGGTGGAATACTTGGAGC
*Gstm4 R*	GCCCAGGAACTGTGAGAAGA
*Gsta4 F*	TGATTGCCGTGGCTCCATTTA
*Gsta4 R*	CAACGAGAAAAGCCTCTCCGT
*Nqo1 F*	AGGATGGGAGGTACTCGAATC
*Nqo1R*	TGCTAGAGATGACTCGGAAGG
*Sod1 F*	AACCAGTTGTGTTGTCAGGAC
*Sod1 R*	CCACCATGTTTCTTAGAGTGAGG
*Sod2 F*	TGGACAAACCTGAGCCCTAAG
*Sod2 R*	CCCAAAGTCACGCTTGATAGC

### Wheat germ agglutinin staining (WGA)

10 μm cryostat sections of atrial tissues from all the experimental animals were fixed in 4 % paraformaldehyde for 15 min and followed by three washes with PBS. Then the tissue sections were labeled with 5 µg/ml of WGA in PBS, wheat germ agglutinin, oregon green 488 Conjugate (W6748, Molecular Probes, Inc.) for 30 min in a light protected chamber maintained at 37 °C incubator for 30 min. Slides were then washed in PBS and fixed with fluoroshield mounting medium with DAPI (ab104139, Abcam, Cambridge, MA, USA) and the images were captured using a confocal laser microscope (Nikon A1Confocal, Nikon Instruments Inc.) using a 60× objective [35].

### Immunofluorescent detection of antioxidants, ubiquitination and autophagy proteins in atria

For the immunofluorescent staining, the atrial tissues collected from all the experimental groups were embedded in O.C.T freezing medium followed by 10 μm sectioning on a cryostat. Tissue sections were incubated with 4 % paraformaldehyde for 15 min and washed thrice with PBS. Then the sections were permeabilized with 0.25 % Triton X-100 and washed thrice with PBS. The tissue sections were then incubated with PBS containing 5 % normal horse serum for 1 h to block nonspecific binding of antibodies. Sections were then incubated with respective primary antibodies for 1 h at room temperature or overnight at 4 °C. The following concentrations for primary antibodies were used: rabbit/mouse anti-GCLC (1:500; ab41463, Abcam, Cambridge, MA, USA); rabbit anti-NQO1 (1:500; ab34173, Abcam, Cambridge, MA, USA), rabbit anti-GSTμ (1:500; ab178684, Abcam, Cambridge, MA, USA), rabbit anti-GSR (1:500; ab16801, Abcam, Cambridge, MA, USA), rabbit anti-4-HNE (1:500; 393207, Calbiochem, EMD Millipore Corporation, Billerica, MA), rabbit anti-Ubiquitin (1:500; ab7780, Abcam, Cambridge, MA, USA), rabbit LC-3 (1:500; D3U4C, Cell Signaling Technology, Danvers, MA, USA), rabbit p62 (1:500; ab91526, Abcam, Cambridge, MA, USA), rabbit ATG-7 (1:500; D12B11,Cell Signaling Technology, Danvers, MA, USA). In some experiments, tissue sections were sequentially double-stained with rabbit anti-ATG-7 primary antibody (1:500; D12B11, Cell Signaling Technology, Danvers, MA, USA) and incubated overnight at 4 °C, followed by 1 h incubation with the secondary antibody Alexa fluor 594 goat anti-rabbit IgG (H+L) (1: 1000; A11037, Life Technologies Corporation, NY, USA) at room temperature for 1 h in dark. Sections were then washed thrice with PBS and incubated with second-primary antibody, rabbit anti-Ubiquitin (1:500; ab7780, Abcam, Cambridge, MA, USA) for 2 h at room temperature. All sections following primary antibody incubation were washed with PBS thrice and then incubated in secondary antibody Alexa fluor 488 goat anti-rabbit IgG (H+L) (1: 1000; A11008, Life Technologies Corporation, NY, USA) for 1 h in the dark. Thereafter, sections were thoroughly washed thrice for 10 min with PBS in dark and fluoroshield/DAPI mounted (ab104139, Abcam, Cambridge, MA, USA) specimens were imaged on a confocal laser-scanning microscope (Nikon A1Confocal, Nikon Instruments Inc.) by using a 60× objective [13]. At least, 3–4 micrographs per tissue were obtained and calculated the appropriate intensity for each target.

### Determination of glutathione (GSH) by immunofluorescence

For the anti-GSH immunofluorescent staining, the atrial tissues collected from all the experimental groups were embedded, fixed and permeabilized as above. Sections were then incubated in ethanol containing 10 mM N-Ethylmaleimide (NEM) [36] for 30 min and washed three times with PBS. After blocking with 1 M PBS containing 5 % normal horse serum for 1 h, sections were incubated for overnight at 4 °C with primary antibody (mouse anti-Glutathione: N- Ethylmaleimide; 1:500; MAB3194, EMD Millipore Corporation, Billerica, MA). After appropriate staining with secondary antibody conjugated with Alexa-Flour-488, sections were then mounted and imaged using a confocal microscope.

### Measurement of ROS production using DHE staining

The level of ROS was measured using a lipophilic, cell-permeable fluorogenic dye, DHE (di-hydro ethidium), which undergoes oxidation to ethidium bromide or structurally similar products in the presence of superoxide that binds with double stranded DNA yielding a red fluorescent signal [37]. Briefly, atria were collected from the different groups and were placed in O.C.T. medium and frozen in −80 °C. 10 μm thick atrial sections were placed on slides and incubated with 5 μg/ml of DHE in PBS in a light protected chamber maintained at 37 °C incubator for 30 min. Slides were then fixed with fluoroshield mounting medium with DAPI (ab104139, Abcam, Cambridge, MA, USA) and the images were captured by randomly selecting 3–5 fields/section were obtained using a Nikon A1 confocal microscope (Nikon Instruments Inc.) and 60× objective. The pixel densities of the digitized fluorescent images were quantified using ImageJ (NIH). Relative changes of DHE fluorescence were expressed as fold change over WT sedentary group.

### Statistical analysis

Mean values for relative gene expression and relative fold change for protein level were calculated and the data points were expressed as mean ± SEM for n  = 3–4 in each experimental group. Statistical analysis for the comparison between the treatment and control groups was performed by one-way ANOVA using Graph-Pad Prism software. Statistical significance was indicated where P < 0.05.

## Results

### Genetic ablation of Nrf2 promotes hypertrophy associated remodeling of the atria in late middle-aged equivalent mice

**Fig. 1:**
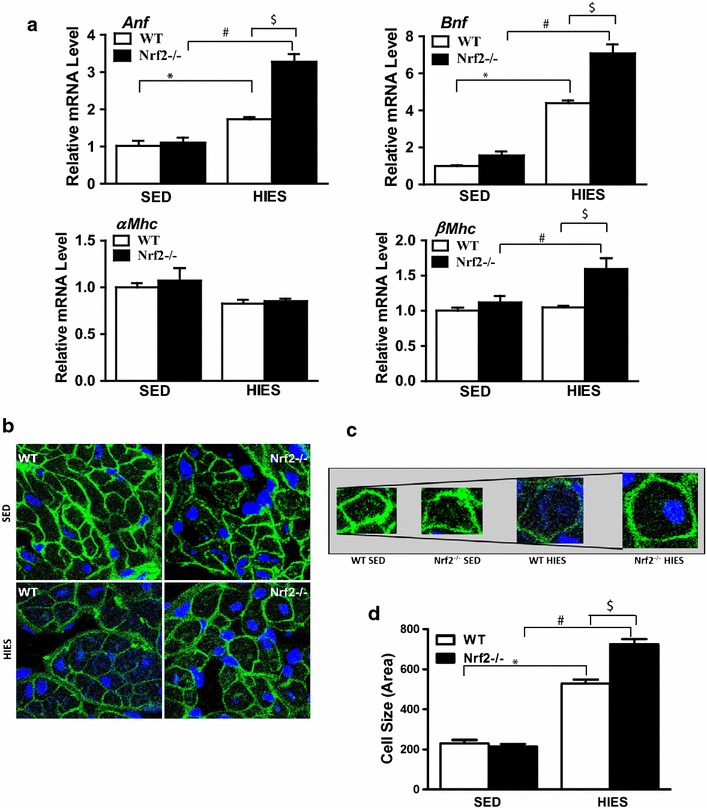
Analysis of gene expression and hypertrophy in the atria of WT and Nrf2^−/−^ mice upon HIES. **a** After 4-weeks of high intensity endurance exercise (HIES), atria were isolated from SED and HIES (n = 4–6/group) mice. Transcript (mRNA) levels of hypertrophy markers (*Anf*, *Bnf*, α-*Mhc* and β-*Mhc*) by qRT-PCR was analyzed and relative gene expression was calculated using Gapdh as a house-keeping gene. **b** Immunofluorescence images showing atrial hypertrophy in HIES WT and Nrf2^−/−^. Cryo-sections from atrial tissues were fixed and stained with wheat germ agglutinin (WGA) and imaged using confocal microscopy (60 × oil immersion). **c** WGA staining showing the *borders* of atrial cardiomyocytes with cell membrane in *green* and DAPI stains nucleus in *blue*-enlarged (hypertrophy) cardiomyocytes are highlighted. **d** Cell size was quantified by measuring area using ImageJ software (n = 30–45 cells/group). Data is presented in the *histograms* (mean ± SEM). *P < 0.05 vs SED WT; ^#^P < 0.05 vs SED Nrf2^−/−^ and ^$^P < 0.05 vs HIES WT

Nrf2-antioxidant signaling declines with advancing age [18, 38] and the mice used herein are between 20 and 22 months, the human equivalent of 60+ years representing late middle age. The significant finding of the current study is that the loss of Nrf2 in conjunction with a high intensity exercise stress (HIES) perturbation induced significant changes in atrial remodeling. After 6-weeks of HIES, the mRNA levels of key markers of hypertrophy (*Anf, Bnf*, α-*Mhc* and β-*Mhc*) were significantly (P < 0.05) altered in atria of Nrf2^−/−^ in relation to WT mice. In sedentary mice, the gene expression of these markers was comparable between the WT and Nrf2^−/−^ atria. On the other hand, there was a notable difference in the animals that were subjected to HIES. A significant transcript abundance of *Anf* (~80 %; P < 0.05), *Bnf* (75 %; P < 0.05) and β-*Mhc* (50 %; P < 0.05) was observed in Nrf2^−/−^ when compared to WT mice that underwent HIES (Fig. 1a). Interestingly, while HIES significantly elevated the *Anf* and *Bnf* transcripts in WT animals, the levels of α-*Mhc* and β-*Mhc* were not altered. The greater degree of upregulation of the hypertrophy markers seen in Nrf2^−/−^ mice in response to HIES could possibly indicate an early molecular sign of atrial abnormality. Following an increase in a subset of hypertrophy markers, we next examined if there is any cell size variation of atria using wheat germ agglutinin (WGA) staining that delineates the cell membrane. WGA staining data indicated that there was no obvious change in the cell size of the atrial myocardium of Nrf2^−/−^ mice when compared to the WT under sedentary state (Fig. 1b, left top vs right top and c). However, a clear cell size variation with an increase in large and medium-sized cells was noted in the atria of Nrf2^−/−^ mice compared to wild type littermate subjected to HIES (Fig. 1b, left bottom vs right bottom and c). Image J quantification of cardiomyocyte cross-sectional size of Nrf2^−/−^ subjected to HIES was normalized to WT cells, illustrating significantly larger atrial cell size from Nrf2^−/−^ (P < 0.05; Fig. 1d, column 3 vs 4) and thus, an atrial structural defect. Overall, these results demonstrate that mice lacking Nrf2 when challenged with HIES can undergo unsought atrial remodeling.

### Ablation or decline in Nrf2 downregulates the transcription of major antioxidant genes in response to HIES

**Fig. 2:**
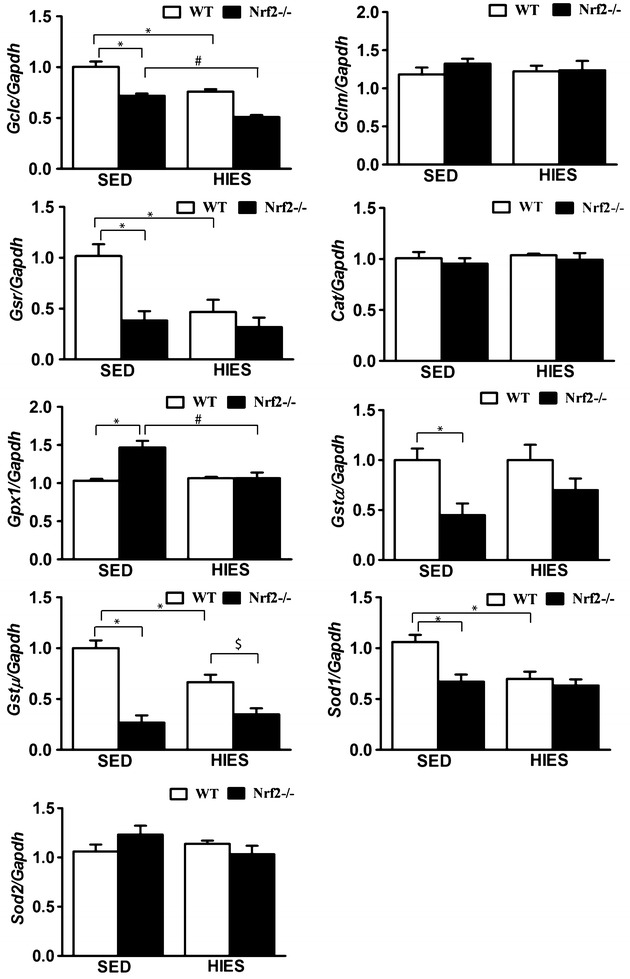
Deregulation of antioxidant genes expression in the atria of WT and Nrf2^−/−^ mice in response to HIES. Quantitative RT-PCR determination of RNA message for Nrf2/ARE-regulated antioxidant genes in the atria of SED and HIES (WT and Nrf2^−/−^, n = 4–6/group) mice. The respective Ct values were normalized to the Gapdh expression and the relative differences in target gene level were determined using the arithmetic formula 2^−ΔΔCt^. The results are presented as mean ± SEM of WT-sedentary. *P < 0.05 vs SED WT; ^#^P < 0.05 vs SED Nrf2^–/–^ and ^$^P < 0.05 vs HIES WT

Under the sedentary condition, the atrial mRNA expression for *Gclc*, *Gsr*, *Gst-µ*, *Gst-α* and Sod1 were moderately down-regulated in Nrf2-knockout versus WT mice (Fig. 2), suggesting the expected decline of antioxidants transcription due to advancing age [38]. In response to HIES, the mRNA levels of *Gclc*, *Gsr*, *Gst-µ*, *Gst-α* and *Sod1* were significantly down-regulated in both the groups indicating decreased or blunted trans-activation of Nrf2-dependent antioxidant genes (Fig. 2). Interestingly the degree of down-regulation in some of the genes (*Gsr*, *Gst-µ*, *Gst-α*) was magnified in Nrf2^−/−^ when compared to WT mice, which reveals the selective loss-of-function effect for Nrf2. Nevertheless, these observations suggest that the WT mice at the age of >20 months display compromised antioxidant systems in response to HIES which could be greatly affected by an absence of Nrf2 as reported previously [32].

### Impaired antioxidants protein expression is evident in Nrf2^−/−^ mice atria under sedentary and/or HIES

**Fig. 3:**
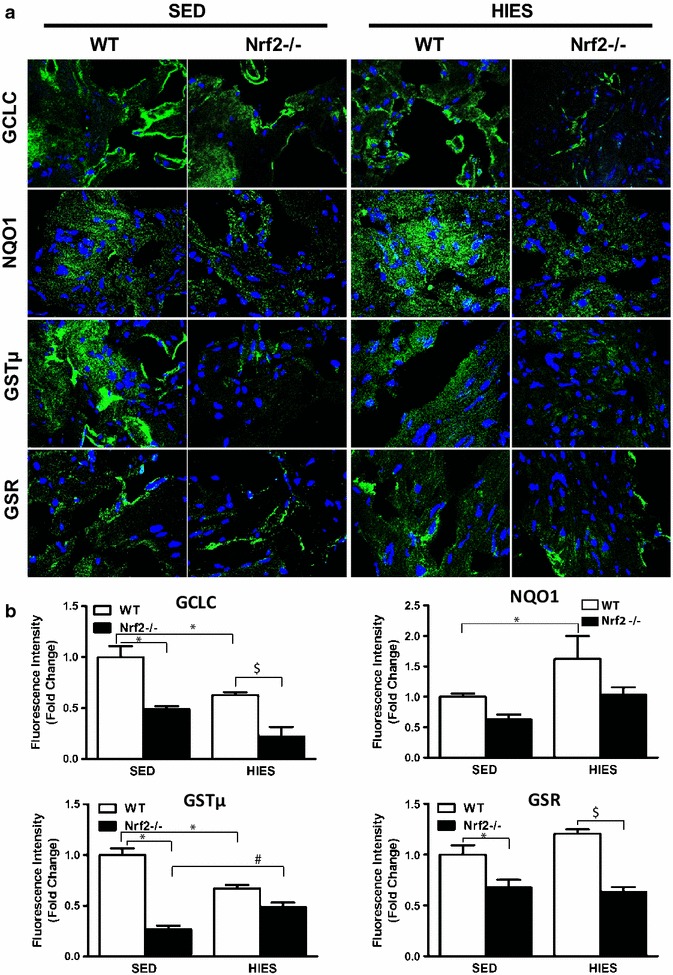
Immunofluorescence analyses of the antioxidant enzymes in the atria of WT and Nrf2^–/–^ mice.** a** IF analysis of representative antioxidant enzymes (GCLC, NQO1, GSTµ and GSR) from cryo-sections of atria from WT and Nrf2^–/–^ mice (n = 3 – 4/group) under sedentary and after HIES.** b** Representative fluorescence intensity analyses of the immune-reactive signals obtained for the respective antioxidant enzymes. *P < 0.05 vs SED WT; ^#^P < 0.05 vs SED Nrf2^–/–^ and ^$^P < 0.05 vs HIES WT

To test if the changes in Nrf2 target gene expression are reflected at the protein levels, we analyzed protein expression of selected antioxidant enzymes that are involved in GSH metabolism and redox homeostasis using immunofluorescence. The tested antioxidant proteins, such as GCLC (1.0 vs 0.5 in Nrf2^−/−^), NQO1 (1.0 vs 0.62 in Nrf2^−/−^), GSR (1.0 vs 0.67 in Nrf2^−/−^) and GST-µ (1.0 vs 0.25 in Nrf2^−/−^) were significantly (P < 0.05) decreased in the atria of >20–22 months old Nrf2^−/−^ mice when compared to age-matched WTs under a sedentary condition (Fig. 3a, b). In addition, HIES challenge to WT animals significantly decreased (GCLC and GSTµ) or increased (NQO1 and GSR) the expression of the selected antioxidant proteins in atria (P < 0.05) when compared to WT sedentary animals (Fig. 3a, b, column 1 vs 3). These observations indicate a differential response in regulating antioxidant proteins by HIES in aged WT mice. Akin to the changes observed in gene expression, the expression of GCLC was further decreased in the atria of Nrf2^−/−^ mice in response to HIES when compared with WT-HIES counterparts (Fig. 3a, b), suggesting that the effect of HIES is exacerbated in the absence of Nrf2. Further, such a dramatic decrease in response to HIES in Nrf2^−/−^ atria might be responsible for regulation of the intracellular glutathione levels.

### Impaired intracellular glutathione levels in Nrf2^−/−^ and HIES mouse atria

**Fig. 4:**
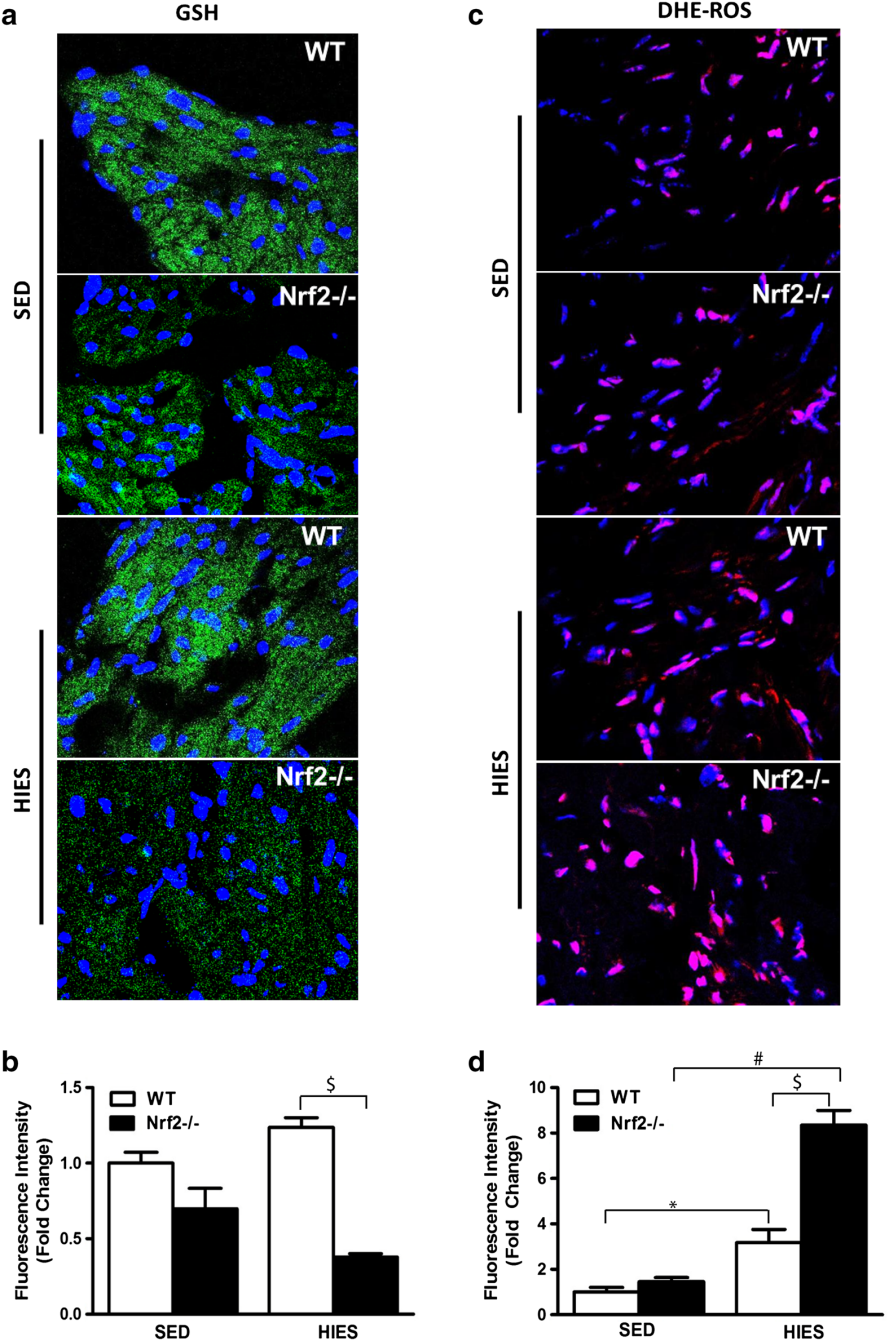
Quantification of reduced glutathione (GSH) and ROS levels by DHE by immunofluorescence: **a** IF analysis using anti-GSH-NEM adduct-ab (1:500; v/v) showing decreased GSH levels in WT and Nrf2^−/−^ under sedentary and after HIES. *Blue*: nucleus (DAPI); *Green*: Anti-GSH staining. **b** Intensity analysis of the Anti-GSH staining. **c** Atrial tissue sections treated with cell-permeable DHE, the oxidized, fluorescent 2-hydroxyethidium (*Red*: DHE; *Blue*: DAPI). *Images* were obtained using confocal microscope at a magnification of 60x oil immersion. **d** ROS intensity was quantified using ImageJ Software and calculated as fold change of mean values of the WT sedentary group and the data is represented as mean ± SEM in the histograms (n = 3/group). *P < 0.05 vs SED WT; ^#^P < 0.05 vs SED Nrf2^−/−^ and ^$^P < 0.05 vs HIES WT

Since we observed a proportional decrease in the levels of GCLC, a GSH biosynthetic enzyme, and GSR, a GSH recycle enzyme in Nrf2^−/−^ mice subjected to HIES, we next examined the levels of GSH under the same experimental setup. In sedentary conditions, the NEM-incubated atrial sections from WT animals showed a mild to moderate level of staining of GSH-NEM (Fig. 4a) that was not significantly different in Nrf2^−/−^ atria (Fig. 4a, b), suggesting the levels of reduced form of GSH is unaffected in the unstressed condition. On the other hand, the Nrf2^−/−^ animals subjected to HIES showed a dramatic reduction in GS-NEM staining. In particular, when compared to HIES challenged WT animals, a clear and significant reduction by ~60 % in the atrial GSH-NEM staining pattern was noted in HIES challenged Nrf2-deficient mice (P < 0.05; Fig. 4b, column 3 vs 4) implying that the reserve of reduced GSH could be profoundly impaired during a stress challenge in the absence of Nrf2 and advancing age.

### Loss of Nrf2 magnifies oxidative stress in response to HIES

**Fig. 5:**
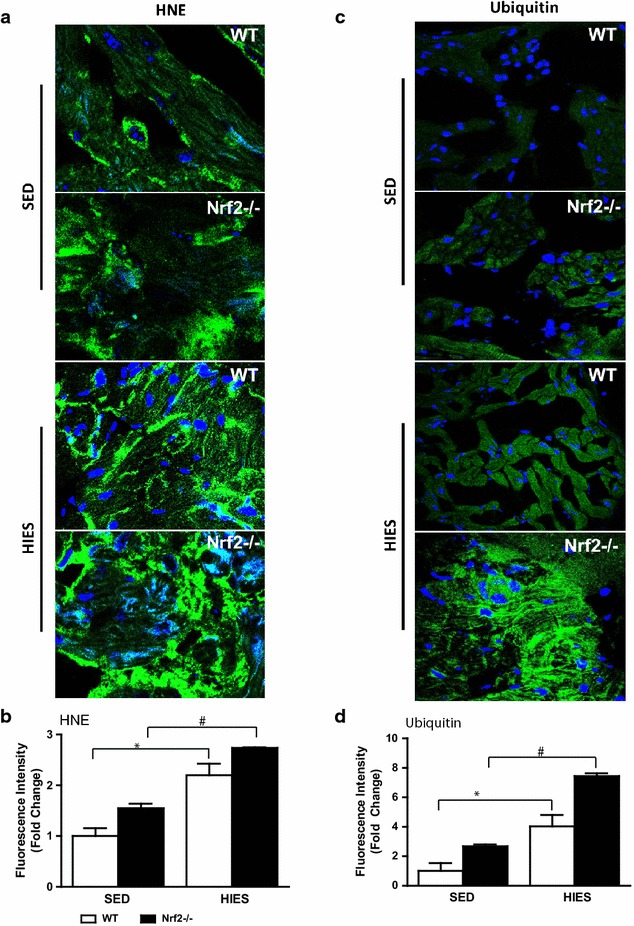
Determination lipid peroxidation by 4-HNE-fluorescence analysis and poly-ubiquitination in WT and Nrf2^−/−^ mice. **a** IF analysis using anti-4HNE-ab (1:500; v/v) showing increased HNE adducts in WT and Nrf2^–/–^ mice. *Blue*: nucleus (DAPI); *Green*: Anti-4HNE staining. **b** Anti-4HNE intensity was calculated as fold change of mean values of the WT sedentary group and the data is represented as mean ± SEM in the histograms (n = 3–4/group). **c** IF of poly-ubiquitination in WT and Nrf2^−/−^ mice. Images were obtained under confocal microscope at a magnification of 60x oil immersion. *Blue*: nucleus (DAPI); *Green*: Ubiquitinated proteins. **d** Intensity analysis of ubiquitinated proteins showing ~ fivefold increase in Nrf2^−/−^ subjected HIES when compared to WT HIES or WT-sedentary mice (n = 3–4/group). *P < 0.05 vs SED WT; ^#^P < 0.05 vs SED Nrf2^–/–^ and ^$^P < 0.05 vs HIES WT

Next, we evaluated whether the decrease in GSH after HIES in Nrf2^−/−^ animals accompany an oxidant load using a small molecule probe, di-hydro ethidium (DHE). DHE staining revealed that WT and Nrf2^−/−^ ablated atria was not significantly different under the non-stressed sedentary state (Fig. 4c, d). However, over 30 % increase in atrial DHE staining was observed in WT animals exposed to HIES (Fig. 4c, d, column 1 vs 3) indicating an increase in reactive oxygen species (ROS) levels. Further, a doubled increase in superoxide levels marked by DHE staining was observed in Nrf2-deficient HIES animals versus WT-HIES (Fig. 4d, column 3 vs 4) illustrating that HIES could perturb and tilt the redox balance towards severe oxidative stress in atria when Nrf2 is abrogated. ROS-induced phospholipid peroxidation can lead to the formation of HNE that in turn can form stable covalent protein adducts and aggravate the pathological processes in the cardiovascular system [39]. As shown in Fig. 5a, under sedentary conditions, atria from Nrf2^−/−^ mice displayed a significantly higher level of 4HNE-Michael adducts when compared to WT animals suggesting that Nrf2 do modulate basal HNE modification of proteins due to advancing age. In addition, HIES exposure to WT animals resulted in a relatively moderate, yet significant increase in HNE-Michael adducts when compared to WT-sedentary animals (Fig. 5b, column 1 vs 3). On the other hand, immunostaining of atrial sections from HIES-challenged Nrf2-knockout mice revealed significantly higher levels of HNE-modified protein adducts (2.73 ± 0.09) than the age-matched WT counterparts (2.19 ± 0.22) (Fig. 5a, b, column 3 vs 4) indicating a positive correlation of HNE-Michael adducts formation along with ROS induction in the atria.

### HIES promotes accumulation of ubiquitinated proteins in the Nrf2^−/−^ atria

The ubiquitin system helps maintain a protein quality control (PQC) and a tight cross-talk exists between ubiquitin and redox homeostasis [40]. As cardiomyocyte size is a tightly regulated process comprising of balanced anabolic and catabolic events, any perturbations in ubiquitin pools and the related pathway could have profound effects on either accrual or removal of key proteins thereby affecting the structure as well as function. Thus, we sought to determine if there are any changes in ubiquitin levels in our experimental conditions using an antibody that detects both free and conjugated ubiquitin in an immunofluorescence assay. Under sedentary conditions, we did not observe any significant staining pattern of ubiquitin immunoreactivity in atria among WT, but Nrf2^−/−^ animals displayed low–moderate levels of ubiquitinated proteins (Fig. 5c, d). While we observed a significant increase in the ubiquitin reactivity in WT mice after HIES, it was not significantly different from the basal Nrf2^−/−^ atria (Fig. 5d). Notably, in a HIES model, Nrf2-knockout exhibited an intense ubiquitin staining of atrial tissue that is largely confined to the cytoplasm, with negligible to nil nuclear staining when compared with WT (Fig. 5c, d). The average atrial ubiquitin staining score of Nrf2^−/−^ underwent HIES (7.43 ± 0.2) was significantly higher than that of the corresponding WT mice (4.03 ± 0.77) (Fig. 5d). Altogether, this data indicated that pools of ubiquitin and its conjugated proteins are higher in atria of Nrf2-disrupted mice subjected to HIES, and its abundance may be associated with increased vulnerability of atrium to oxidative injury mediated structural remodeling.

### Impaired autophagy response is evident in Nrf2^−/−^ after HIES

**Fig. 6:**
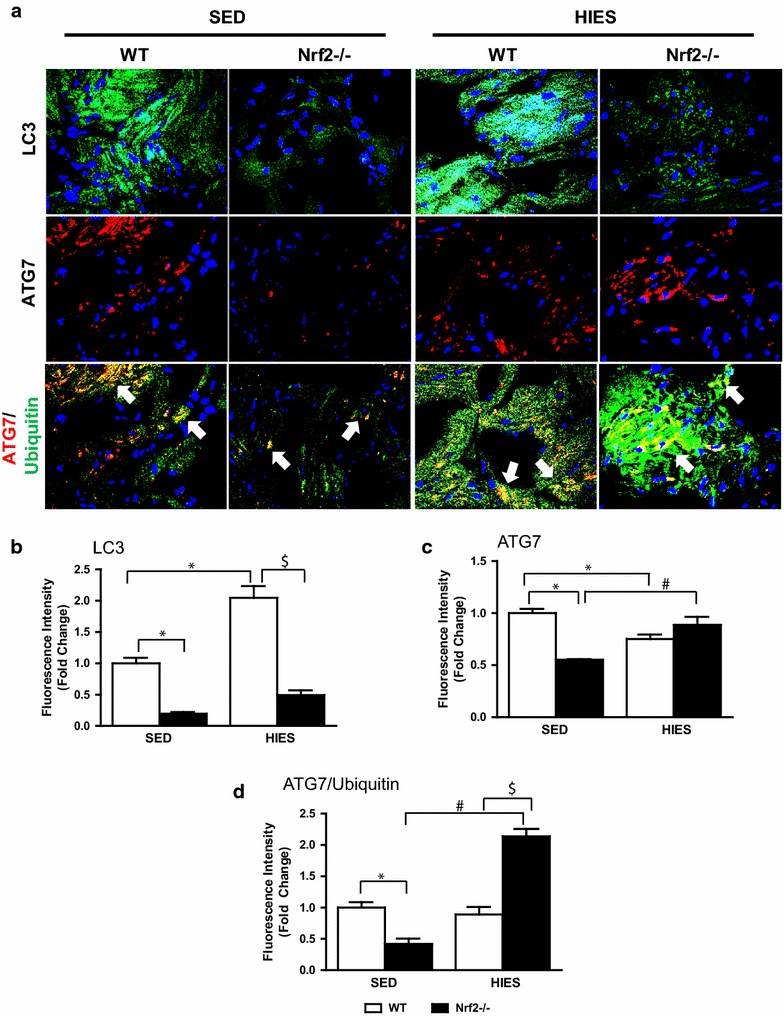
Determination of autophagy proteins (LC3 & ATG7) in WT and Nrf2^−/−^ in response to HIES. **a** IF analysis of autophagy antibodies LC3 (1:500; v/v) and ATG7 (1:500; v/v) and double immunostaining of ATG7/Ubiquitin (1:500; v/v). Blue: nucleus (DAPI); Green: localizes LC3; *Red*/*Green* merge (*Yellow*): ATG7/Ubiquitin. **b** Fluorescence intensity was calculated as fold change of mean values of the WT sedentary group and the data is represented as mean ± SEM in the histograms (n = 3–4/group). *P < 0.05 vs SED WT; ^#^P < 0.05 vs SED Nrf2^−/−^ and ^$^P < 0.05 vs HIES WT

Due to increased ubiquitination in response to HIES and since the Nrf2 pathway has been reported to play a critical role in autophagy mechanism, we next sought to evaluate the status of autophagy in this setting. The impact of HIES on atrial autophagic activity in the WT and Nrf2^−/−^ mice was evaluated by determining the expression of LC3, a marker of autophagosome [41], and Atg7, a critical rate limiting factor for autophagosome formation [42]. Immunofluorescence analysis revealed that there was a significant decrease of LC-3 staining in the Nrf2^−/−^ mice under sedentary when compared to WT animals (Fig. 6a). After HIES stimulation, the LC3 levels were significantly increased in WT animals, but this response was less pronounced in Nrf2^−/−^ atria (Fig. 6a, b). An increasing response of ATG7-positive structures was noted in atria of Nrf2^−/−^ after HIES when compared to Nrf2^−/−^ sedentary mice (Fig. 6a, c) implying activation of the autophagy process. Further, double immunostaining of ATG7/ubiquitin revealed an extensive cytoplasmic co-localization of ubiquitin and ATG7 on both large pleomorphic and small puncta structures along with increased number of ubiquitin/ATG7 double positive counts in Nrf2^−/−^ mice challenged with HIES when compared with WT that underwent HIES (Fig. 6a, d; white arrows (lower panel). In contrast, they were either barely or mildly detected under the sedentary state. Altogether, these results suggest that the loss of Nrf2 can exaggerate HIES-impaired autophagosome formation and result in accumulation of toxic proteins in the atria.

## Discussion

**Fig. 7:**
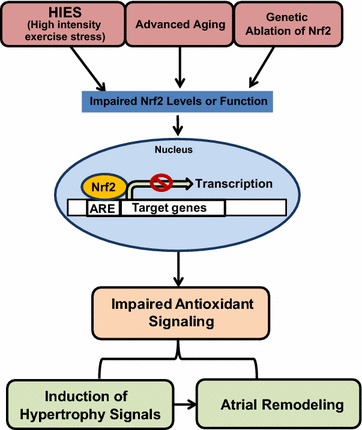
Proposed model displaying the effects of high intensity endurance stress (HIES) on atrial remodeling on aging and/or under Nrf2 ablation

The atrium is a dynamic organ that instigates electric signals to the ventricle to successively beat and facilitate blood circulation to peripheral organ systems. While redox homeostasis is critical for the regulation of basal cellular signaling events, its shift towards oxidative stress along with an impaired antioxidant system is expected to be detrimental and leads to end-organ damage. The atrium being such an important organ system, its exposure to oxidative stress is shown to structurally and electrically remodel and result in atrial fibrosis and/or atrial fibrillation. In the present study, we investigated whether genetic ablation of the master regulator of antioxidants namely, nuclear erythroid derived factor-2 (Nfe2L2 or Nrf2) could induce pathological atrial remodeling in a human equivalence of late middle-aged mice undergoing high intensity exercise stress (HIES). Our findings indicate that (i) loss of this transcription factor results in differential regulation of antioxidant systems in the atria, (ii) HIES induces adaptive hypertrophy response in WT, but the Nrf2-null mice exhibit pathological atrial remodeling, (iii) WT mice maintains glutathione levels after HIES, but Nrf2^−/−^ exhibit depletion of GSH and (iv) profound oxidative stress observed in Nrf2-null mice subjected to HIES was associated with increased ubiquitination resulting in impaired autophagic mechanisms. Altogether, our results suggest a critical role for Nrf2 in maintaining the gene indices responsible for optimal atrial efficiency in response to HIES (Fig. 7).

Hypertrophy is accompanied by changes in expression of specific genes [43–45]. In the 20–22 months old WT mice, HIES significantly induced the expression of hypertrophic markers, *Anf* and *Bnf* with no change in α-*Mhc* (Myh6) and β-*Mhc* (Myh7) expression. On the other hand, ablation of Nrf2 along with high intensity exercise resulted in a several fold increase in the transcript levels of markers of hypertrophy (Fig. 1). Surprisingly, the β-*Mhc* expression was significantly increased without a change in α-*Mhc* in response to HIES in the Nrf2^−/−^ atria. This result is in accordance with the previous reports that upon atrial hypertrophy, MHC isozyme undergo a transition from α-MHC to β-MHC [46–48]. Furthermore, the differential MHC isoenzyme distribution observed in Nrf2^−/−^ mice subjected to HIES could indicate a pathological structural remodeling of the atria as reported in various pathological conditions such as diabetes, dwarfism, adrenalectomy and gonadectomy [49–53].

A progressive decline in overall antioxidant mechanisms is strongly associated with profound oxidative stress with advancing age, which is believed to be a causal factor for various pathological processes including cardiovascular diseases. Although several studies have reported a strong role for Nrf2 in various organ systems [15, 38], there is no information available on its role in the atrial tissue. Current observations show evidence for impaired antioxidant mechanisms due to the loss of Nrf2 in the atria. Under unstressed conditions, downregulation of some of the antioxidants at the level of transcription and translation was noted in association with ablation of Nrf2 in late middle-aged (20–22 months) mice. Interestingly, sustained strain created by HIES intensified the impairment of selective antioxidant genes/proteins in atria due to Nrf2 ablation. Nonetheless, the atria of WT animals found to either retain or partially induce the transcription/translation of some of the antioxidants (GSR and NQO1) in response to HIES. These observations in WT mice recalls our previous finding that acute exercise activating Nrf2-antioxidant signaling in young-adult mice hearts [25]. Similar studies related to active versus sedentary life style in humans indicated impaired activation of Nrf2-signaling in aged skeletal muscle [18, 38]. Here, the genetic ablation of Nrf2 combined with HIES significantly but selectively impacted the antioxidant system in atria plausibly due to switching and/or availability of co-factors controlling the transcriptional events which are stressor-specific that influences distinct pathways and regulatory factors [54].

Oxidative stress coupled with advancing age is believed to cause a variety of biochemical and conformational alterations in macromolecules [55–57]. Under a chronic setting, these changes along with prolonged antioxidant depletion in organelles and cells result in impaired PQC and organ dysfunction. Several studies have reported that increased ROS/RNS generation and oxidative/nitrosative stress contribute to several age-associated diseases including cardiovascular complications in humans [9, 44, 52, 58, 59]. Declined Nrf2 signaling is associated with increased oxidative stress in the skeletal muscle of sedentary humans when compared to active humans with advancing age [60]. Here, 20–22 months old WT mice displayed increased oxidative stress and impaired antioxidant system in atria after HIES, that was further magnified in the Nrf2^−/−^ mice due to the loss of inducible cytoprotective mechanisms. Maintenance of moderate levels of antioxidant system components is central to govern the intracellular oxidant load and protect the cells/organs from oxidative stress [32]. In the current study, alteration of redox homeostasis due to Nrf2 abrogation enhanced the extent of atrial ubiquitination in response to HIES. In addition, we found that 4-HNE modified proteins, end products of lipid peroxidation were significantly elevated in HIES-subjected Nrf2-null mice when compared with its WT counterparts. Since high levels of ubiquitination and 4-HNE are reported to induce cardiac hypertrophy, cellular damage and apoptosis in the heart [61–66], the significantly elevated levels of 4-HNE modified proteins along with ubiquitination that we found after HIES in Nrf2-null animals may result in pathological atrial hypertrophy. Therefore, a balanced action of Nrf2 signaling is vital to preserve atrial antioxidant defense and redox homeostasis.

While reduced autophagy has been related to accelerated aging, activation of autophagy might have potent anti-aging effects [67, 68]. Interestingly, while exercise stress activates autophagy response (LC3) in WT and Nrf2^−/−^ atria, the degree of accumulation of ubiquitinated proteins is significantly increased in the Nrf2^−/−^ when compared to WT mice in response to HIES. In particular, co-staining of ATG7 with poly-ubiquitin-antibody (Fig. 6a,d) suggests its ubiquitination indicating an impairment in autophagy function in the Nrf2^−/−^ atria. ATG7 is a key rate limiting component of autophagy pathway that regulates the formation of autophagosome for further presentation to the lysosome to form the autophagolysosome [69]. Besides autophagy being an adaptive response to stress and maintain PQC in cells, it has been reported to have a prominent role in defining the life span of many model organisms [70]. Of note, under HIES setting, a higher degree of impairment in autophagic response (due to ubiquitination of ATG7) observed in Nrf2^−/−^ in relation to WT mice might form the basis for compromised PQC and accumulation of degraded proteins in the atrial myocytes, and may explain the plausible mechanism for pathological remodeling of Nrf2^−/−^ atria. Recently, a unique form of ubiquitin-dependent autophagy in protein aggregate clearance with relevance to multiple pathological setting is increasing [71–73]. Furthermore, our data on the impaired ubiquitination system and autophagic mechanisms due to loss of Nrf2 could complicate the treatment strategies as recently, it has been documented that a collaborative action between autophagy and ubiquitination is essential to target an oncogenic protein for prevention or therapy [74].

## Conclusions

The fundamental observations presented here suggests that loss of Nrf2 could have independent and/or unconventional effects beyond antioxidant control in that it could either directly or indirectly be involved in deregulating the gene indices associated with increased ubiquitination and impaired autophagy leading to atrial hypertrophy. In terms of HIES, WT atria are characterized by mild to a modest increase in genes associated with hypertrophy and antioxidant systems that are controlling structure-function characteristics of atria hinting at a physiological adaptation. However, Nrf2-null atria displayed a greater magnitude of changes in the aforementioned parameters beyond optimal adaptation. Our current study using the whole body Nrf2-KO model emphasizes that the loss of Nrf2 can reduce key cytoprotective proteins and could blunt the protective response to exercise induced-oxidative stress in aged animals thus trigger a pathological remodeling of the atria. In other words, the vital requirement of Nrf2 to obtain the benefits of exercise is mandatory during aging and in addition, manipulating the Nrf2-ARE signaling could likely be a potential means to mitigate atrial maladaptations and damages that occur during age-associated fibrotic remodeling and atrial fibrillation. In conclusion, we propose that loss of Nrf2 could switch the physiological adaptation to pathological phenotype in human equivalence of late middle-aged atria undergoing high intensity exercise stress.
